# Neuroprotective role for RORA in Parkinson’s disease revealed by analysis of post-mortem brain and a dopaminergic cell line

**DOI:** 10.1038/s41531-023-00563-4

**Published:** 2023-07-27

**Authors:** Felwah S. Al-Zaid, Michael J. Hurley, David T. Dexter, Glenda E. Gillies

**Affiliations:** 1 Department of Physiology, College of Medicine, King Saud University, Riyadh, KSA Saudi Arabia; 2 Department of Brain Sciences, Imperial College London, London, W12 0NN UK; 3 Department of Clinical and Movement Neuroscience, UCL Queen Square Institute of Neurology, Rowland Hill Street, London, NW3 2PF UK; 4 Parkinson’s UK, 215 Vauxhall Bridge Road, London, SW1V 1EJ UK

**Keywords:** Parkinson's disease, Diseases of the nervous system, Neurology, Movement disorders

## Abstract

Parkinson’s disease (PD) is almost twice as prevalent in men, which has largely been attributed to neuroprotective effect of oestradiol in women. RORA (retinoic acid receptor-related orphan receptor alpha) regulates the transcription of central aromatase, the enzyme responsible for local oestradiol synthesis, simultaneously, RORA expression is regulated by sex hormones. Moreover, RORA protects neurones against oxidative stress, a key mechanism contributing to the loss of dopaminergic neurones in PD. Therefore, we hypothesized that there would be sex differences in RORA expression in the substantia nigra pars compacta (SNpc), which could contribute to sex differences observed in PD prevalence and pathogenesis. In a case control study, qPCR and western blot analyses were used to quantify gene and protein expression in the SNpc of post-mortem brains (*n* = 14 late-stage PD and 11 age and sex matched controls). The neuroprotective properties of a RORA agonist were then investigated directly using a cell culture toxin-based model of PD coupled with measures of viability, mitochondrial function and apoptosis. RORA was expressed at significantly higher levels in the SNpc from control females’ brains compared to males. In PD, we found a significant increase in SNpc RORA expression in male PD compared to female PD. Treatment with a RORA agonist showed a significant neuroprotection in our cell culture model of PD and revealed significant effects on intracellular factors involved in neuronal survival and demise. This study is the first to demonstrate a sex specific pattern of RORA protein and gene expression in the SNpc of controls post-mortem human brains, and to show that this is differentially altered in male and female PD subjects, thus supporting a role for RORA in sex-specific aspects of PD. Furthermore, our in vitro PD model indicates mechanisms whereby a RORA agonist exerts its neuroprotective effect, thereby highlighting the translational potential for RORA ligands in PD.

## Introduction

Parkinson’s disease (PD) is a progressive neurodegenerative movement disorder characterized by reduced striatal dopamine levels due to degeneration of dopaminergic neurones in the substantia nigra pars compacta (SNpc)^1^. PD is the second most common age-related neurodegenerative disease after Alzheimer’s disease (AD) and the most common movement disorder^2^, and features among the leading causes of death in the United States in 2013^3^. The overall incidence of PD has been estimated to be 12–16^4^ and 16–19^5^ per 100,000 person-years in US and European populations, respectively. The number of world-wide PD cases is estimated to increase from 4.1 million (340,000 US cases) in 2005 to nearly 8.7 million (610,000 US cases) by 2030^6^. Despite decades of research, the principal available therapeutic strategy for PD is dopamine replacement therapy with L-DOPA or treatment with directly acting dopamine receptor agonists to provide symptomatic relief of motor symptoms^7^. However, these treatments can result in disabling adverse side effects (e.g., dyskinesia, psychosis), and lose efficacy in the long term^7^.

After age, male sex is a major risk factor for developing PD, with a biased male-to-female ratio reported to range from 1.3 to 3.7^4,5,8,9^. This sex bias extends beyond incidence to include age of onset, clinical course and presentation of the disease, pathogenesis, and responsiveness to treatments^10–13^. For example, women have a significantly later age of onset of PD than men and exhibit more benign symptoms initially but have a greater risk of developing treatment-related complications^5,11,14–17^.

Although the exact mechanisms underlying the sex bias in PD are unclear, many clinical observations and experimental studies in rodents and non-human primates have identified gonadal sex hormone influences, especially the neuro-protective effect of oestradiol, as playing a key role^10,11,13,18,19^. Interestingly, experimental models of PD revealed that although peripherally circulating oestradiol was neuroprotective in females, there was no such protective effect in males, where oestradiol treatment even exacerbated striatal dopamine loss^11,20^. However, in contrast to the sex differences in the protective influence of peripherally circulating oestradiol, inhibition or knock-down of central aromatase, the enzyme responsible for the local synthesis of oestradiol in the brain, revealed that generation of oestradiol within the nigrostriatal dopaminergic system was neuroprotective in experimental PD in both males and females^21–24^. This indicates that selective regulation of brain aromatase offers unique therapeutic potential for both sexes by increasing the concentration of oestradiol in brain^10^, but a knowledge of potential regulatory factors is lacking.

Notably, transcription of aromatase is regulated by the RORA gene^25^, which is known to have many direct neuronal functions, including protection against oxidative stress-induced neuronal damage, which is a key process in the aetiology of PD^26–28^. RORA was also reported to be neuroprotective in an experimental model of hypoxia^29^ and has been found to be linked to many neurological and psychological disorders, including Alzheimer’s disease, depression, autism, attention deficit hyperactivity disorder, post-traumatic stress disorder, bipolar disorder and fear related psychopathology^30–34^. RORA and its target genes have also been identified as important molecular mediators of the role played by sex hormones in autism susceptibility, which displays a marked male sex bias^25,30,35^. However, a potential involvement of RORA in PD is unexplored.

In the present study, we investigated the expression of RORA in the SNpc of post-mortem brains from patients dying from PD and in age- and sex-matched controls, in order to investigate any potential link to PD and its clinically identified sex bias. Additionally, we adopted a dopaminergic cell culture model of PD in order to investigate directly the potential neuroprotective effects of RORA and its underlying mechanisms, taking advantage of the recently characterised pharmacological tools of the RORα/γ agonist (SR1078) and antagonist (SR1001)^36^. Collectively, our findings support the translational importance of RORA ligands as novel therapeutic agents for PD.

## Results

### Quantification of RORA gene and protein expression in the SNpc and CgCx

**Fig. 1:**
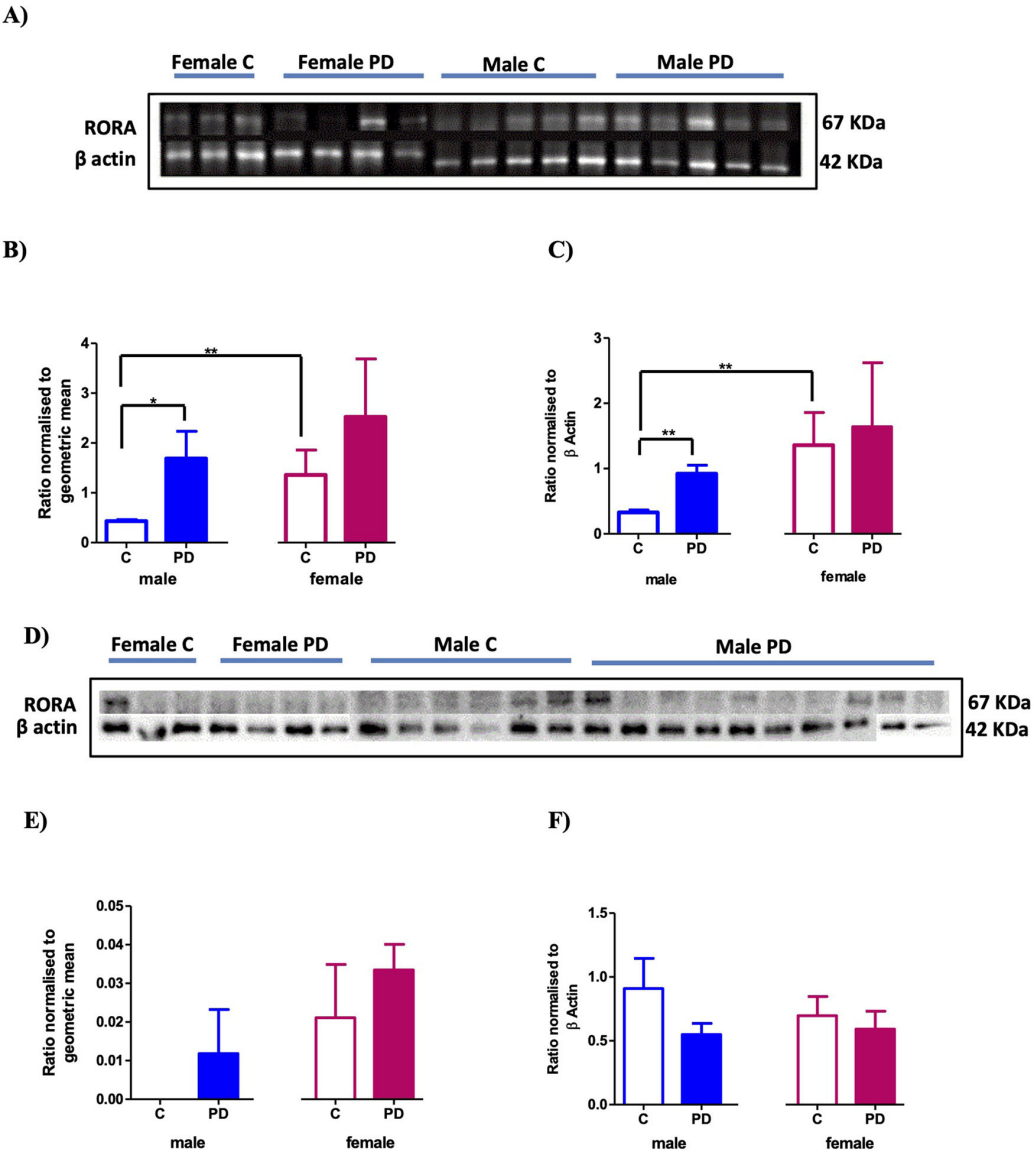
RORA protein and gene expression in the SNpc and CgCx of male and female PD and controls. RORA expression in the SNpc (**A**–**C**) and CgCx (**D**–**F**) of control (**C**) and PD and post-mortem brains. **A** Representative western blot protein bands for RORA in PD and controls SNpc. **B** qPCR analysis of RORA gene expression relative to GAPDH and β actin gene expression (geometric ratio) for males (**C** *n* *=* 5, PD, *n* *=* 9) and females (**C** *n* *=* 6, PD *n* *=* 5) in the SNpc, *p *<* 0.05, **p *<* 0.01 (Mann–Whitney test). **C** RORA protein quantification normalized to β actin for males (**C** *n* *=* 5, PD, *n* *=* 5) and females (**C** *n* *=* 3, PD *n* *=* 4) in the SNpc **P *<* 0.01, (Mann–Whitney test). **D** Representative western blot protein bands for RORA in the CgCx. **E** qPCR analysis of RORA gene expression relative to GAPDH and β actin gene expression (geometric ratio) in the CgCx of the post-mortem brains for males (**C** *n* *=* 5, PD, *n* *=* 9) and females (**C** *n* *=* 5, PD *n* *=* 5). **F** RORA protein quantification normalized to β actin for males (**C** *n* *=* 6, PD, *n* *=* 10) and females (**C** *n* *=* 3, PD, *n* *=* 4) in the CgCx. Mann–Whitney test shows no significant differences. Error bars represent standard error of the mean.

RORA gene expression in the control SNpc was significantly lower, by approximately a third, in males compared to females (*P* < 0.01 and Fig. 1B) (male control: 0.432 ± 0.068, female control: 1.358 ± 1.224). In male PD subjects, RORA SNpc gene expression was significantly greater by more than two-fold compared to controls (*P* < 0.05), while in females, there was no significant difference between the control and PD subjects (male PD: 1.535 ± 1.625, female PD: 2.524 ± 2.602) as shown in Fig. 1B. The pattern of RORA protein expression data in the SNpc closely mirrored the gene expression data. Hence, protein expression levels of RORA in the SNpc in female controls were three-fold greater than in male controls (*P* < 0.01 and Fig. 1A, C) (female control: 1.358 ± 1.118, male control: 0.328 ± 0.077) and in PD this was elevated 2-fold in males relative to controls (*P* < 0.01), but unchanged in females (male PD: 0.922 ± 0.291, female PD: 1.641 ± 1.697) Fig. 1B.

In contrast to the SNpc, western blot analysis of RORA protein expression in the CgCx showed no significant difference between male and female controls and no significant effect of PD in either sex compared to their respective controls (male control: 0.9135 ± 0.236, female control: 0.697 ± 0.156, male PD: 0.552 ± 0.085, female PD: 0.591 ± 0.129) (Fig. 1D, F).

### Cell culture experiments

#### Effect of RORα/γ ligands on 6-OHDA toxicity on N27 cells

**Fig. 2:**
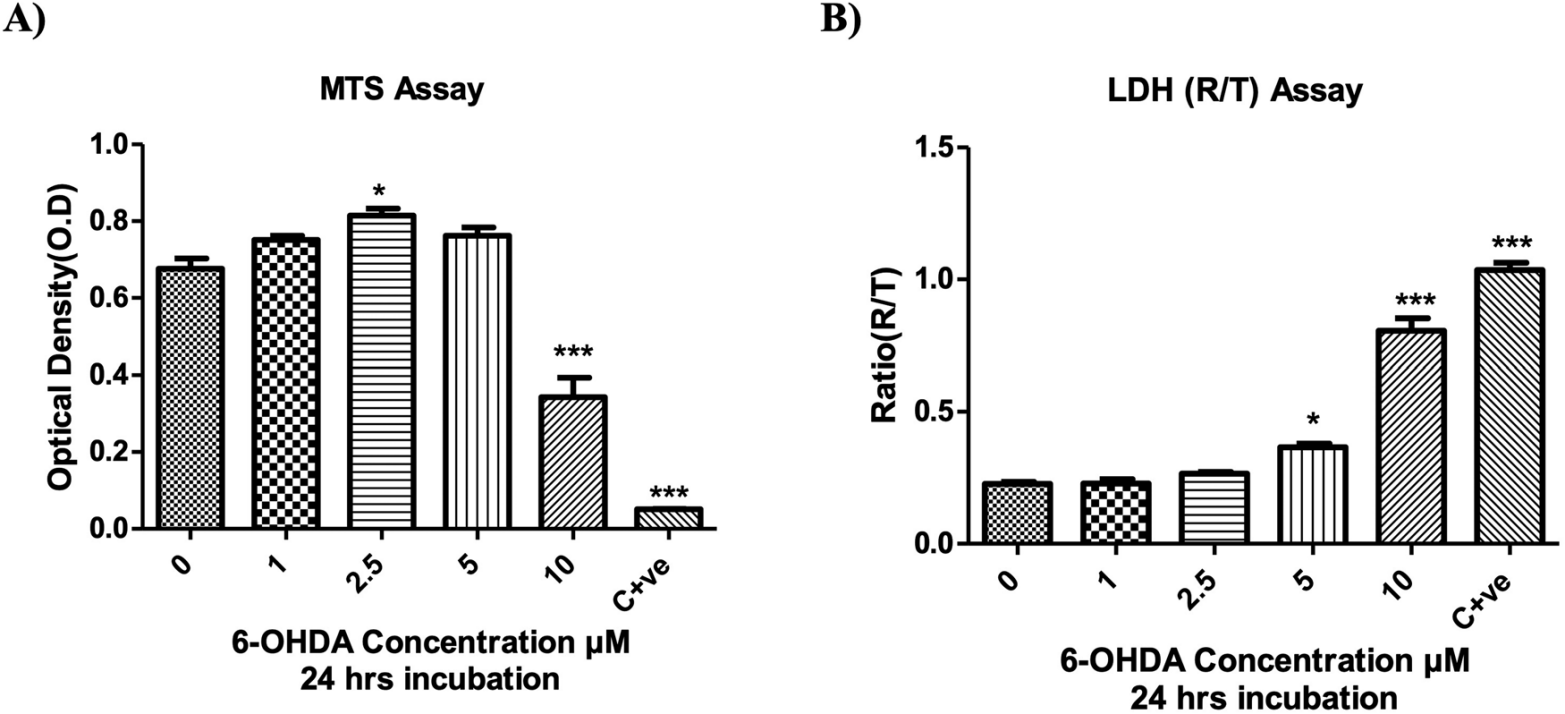
Cytotoxic effect of increasing 6-OHDA doses on N27 cells. **A** MTT assay and **B** LDH assay (released/ total LDH (R/T)) for N27 cells plated at 157 cells/mm^2^, in phenol red free RPMI supplemented with 1% double stripped serum and incubated for 24 h before being treated with increasing 6-OHDA concentrations for 24 h. Positive control (C + ve) was exposure to Staurosporine. Data presented as mean ± SEM of 3 independent experiments, each conducted in duplicate wells. *P* values were generated by one-way ANOVA, Tukey’s *post hoc* tests. *P* values were considered significant when <0.05 and denoted as follows: *P < 0.05, **P < 0.01, ***P < 0.001. Error bars represent standard error of the mean.

The toxic effects of increasing concentrations of 6-OHDA on N27 cells were indicated by the significant, dose-dependent reduction in cell viability, as shown by reduced MTS optical density (Fig. 2A and *P* < 0.001), and increased cell death, shown by increased LDH release (Fig. 2B and *P* < 0.001), compared to unexposed control cells. For subsequent experiments, a concentration of 10 µM 6-OHDA was selected for investigations of potential positive/negative effects of RORα/γ ligands on 6-OHDA-induced toxicity.

**Fig. 3:**
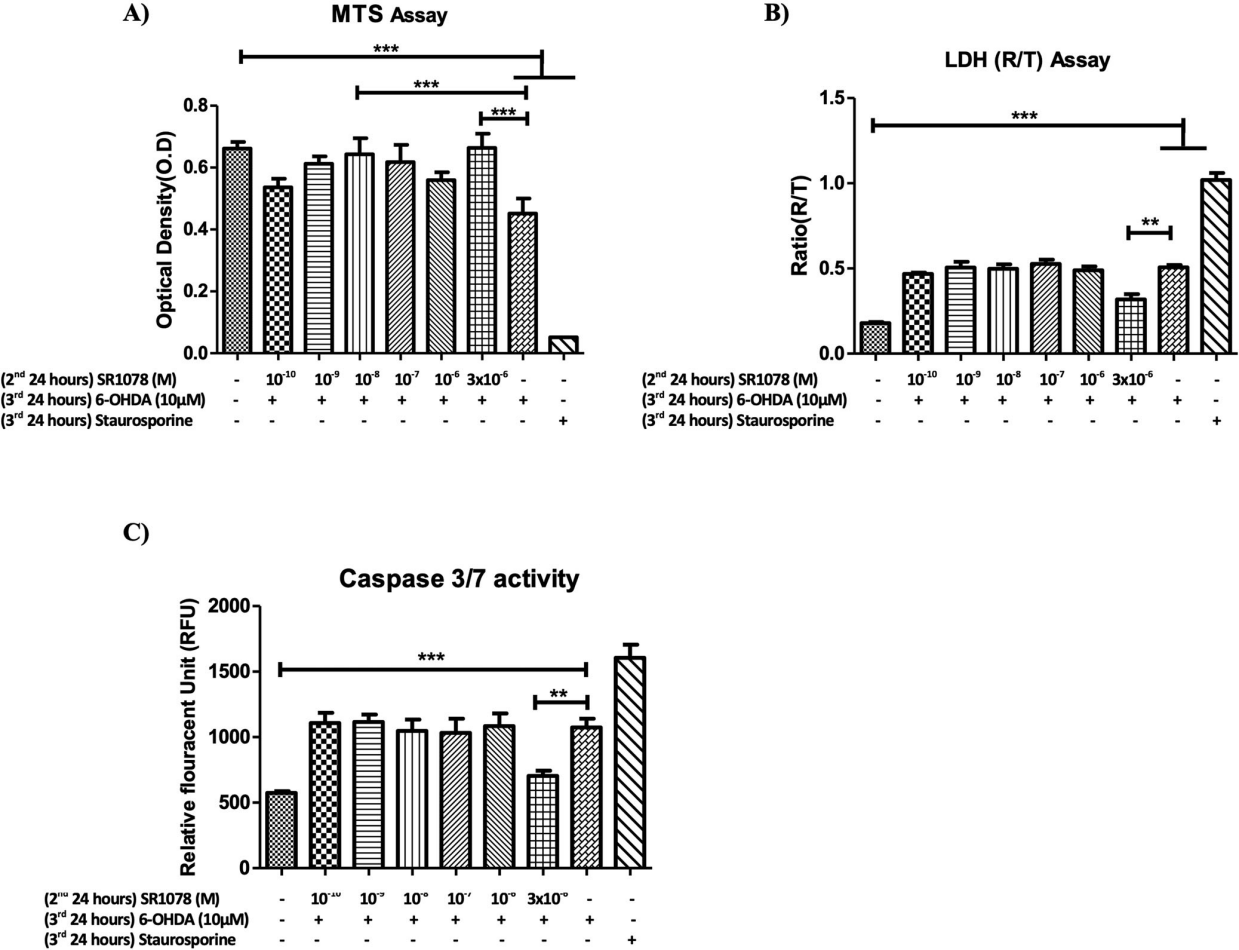
Neuroprotective effect of SR1078 against 6-OHDA on N27 cells. **A** MTS assay (**B**) LDH assay. **C** Caspase 3/7 activity. Cells were plated at density of 157 cells/mm^2^, in RPMI phenol red free medium containing 1% double stripped serum. Cells were allowed to establish for 24 h (1st 24 h) followed by 2nd incubation period for 24 h with SR1078, followed by 24 h’ incubation with 6-OHDA alone. Incubation with Staurosporine alone for the final incubation (3rd 24 h) represents the positive control. Data presented as mean ± SEM of 3 independent experiments, and each conducted in duplicate wells. P values were generated by one-way ANOVA, Tukey’s post hoc tests. *P < 0.05, **P < 0.01, ***P < 0.001. Error bars represent standard error of the mean.

**Fig. 4:**
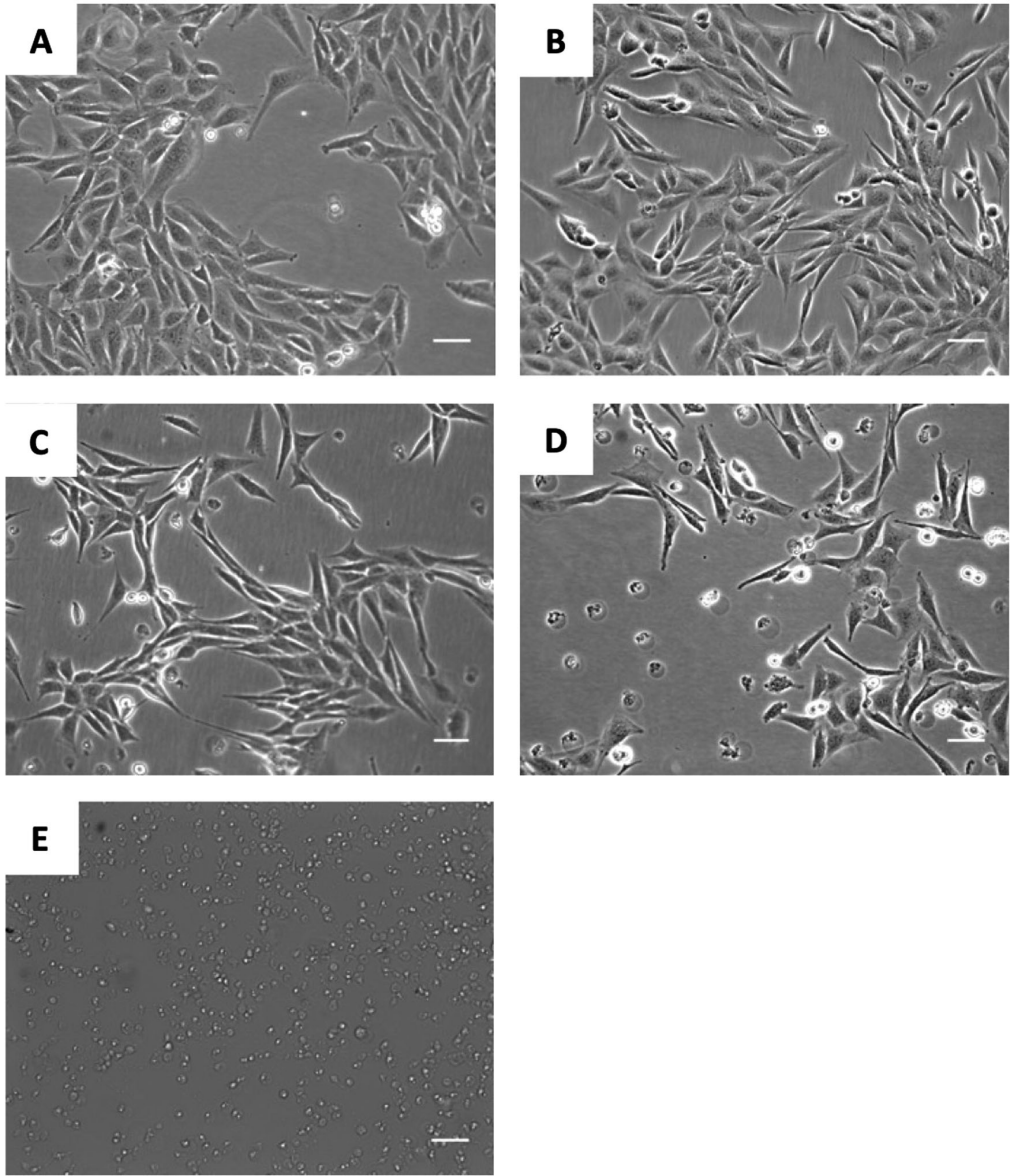
Microscopic images for the neuroprotective effect of SR1078 against 6-OHDA in N27 cells. Cells were plated at initial density of 157 cells/mm^2^ and treated as described for Fig. 4. After the final incubation, cells were imaged using inverted dark field microscopy (**A**) negative control, (**B**) cells treated with SR1078 (3 × 10^−6^ M) for 24 h. **C** Cells treated with SR1078 (3 µM) for 24 h followed by 6-OHDA (10 µM) for 24 h. **D** Cells treated with 6-OHDA (10 µM) alone. **E** Positive control (Staurosporine). Bar represents 50 µm.

**Fig. 5:**
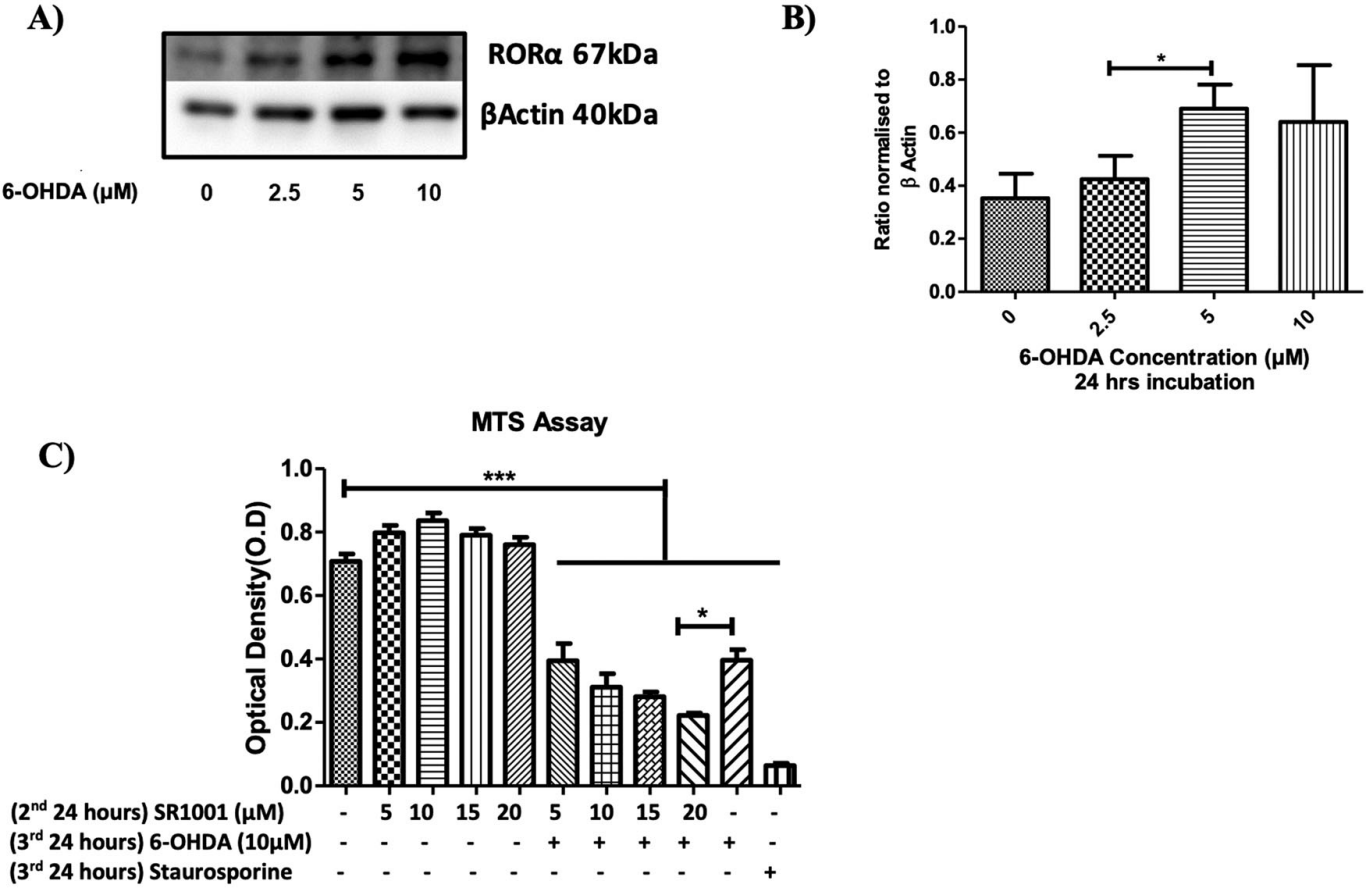
The effect of increasing doses of 6-OHDA on RORA protein quantification in N27 cells and the effect of SR1001 against 6-OHDA on N27 cells. **A** Representative protein bands for RORα western blots. **B** Protein quantification for RORα normalised to β actin in the N27 cells treated with increasing concentration of 6-OHDA (2.5, 5, 10 µM) for 24 h. **C** MTS assay. Cells were plated at density of 157 cells/mm^2^, in RPMI phenol red free medium containing 1% double stripped serum. Cells were allowed to establish for 24 h (1st 24 h) followed by 2nd incubation period for 24 h with SR1001, followed by 24 h’ incubation with 6-OHDA alone. Data presented as mean ± SEM of 3 independent experiments, and each conducted in duplicate wells. *P* values were generated by one-way ANOVA, Tukey’s post hoc tests. *P* values were considered significant when <0.05 and denoted as follows: *p < 0.05, **p < 0.01, ***p < 0.001. Error bars represent standard error of the mean.

Figures 3–5 show that treatment of the cultures with a range of concentrations of either the RORA agonist (SR1078) or antagonist (SR1001) alone had no effects on the parameters tested. Figure 3 shows that the toxic effects of 10 µM 6-OHDA, as demonstrated in the MTS assay (Fig. 3A; *P* < 0.01) and the LDH assay (Fig. 3B; *P* < 0.001), were significantly attenuated when the cells were pre-treated with 3 µM SR1078. Additionally, 6-OHDA significantly enhanced caspase 3/7 activity (Fig. 3C; *P* < 0.001) compared to unexposed cells, indicating an involvement of apoptotic mechanisms, whereas pre-treatment of the cells with 3 µM SR1078 significantly blocked this effect (Fig. 3C; *P* < 0.001). The neuroprotective effects of 3 µM SR1078 reported in Fig. 3 were corroborated when the cell cultures were visualised under inverted dark field microscopy. The images confirm that the appearance of cells treated with SR1078 alone (Fig. 4B) was identical to untreated controls (i.e., no effect) and that the clear cell destruction caused by 6-OHDA (Fig. 4D) was attenuated by pre-treatment of the cultures with 3 µM SR1078 (Fig. 4C).

Figure 5A, B shows that N27 cells themselves can synthesise RORA protein and that these levels are significantly increased after exposure to 6-OHDA. A potential role for the endogenously synthesised RORA was then tested by treating the cultures with the RORA antagonist prior to 6-OHDA. Figure 5C demonstrates that N27 cell death induced by exposure to 6-OHDA (10 µM) was significantly exacerbated by pre-treatment of the cells with 20 µM SR1001, as indicated in the MTS assay.

### Potential mechanisms of SR1078 protection against 6-OHDA toxicity

A panel of factors known to participate in intracellular molecular cascades involved in cell death or survival were investigated in N27 cells that were untreated, exposed to SR1078 alone or to 6-OHDA with or without pre-treatment of SR1078.

#### Protein quantification

**Fig. 6:**
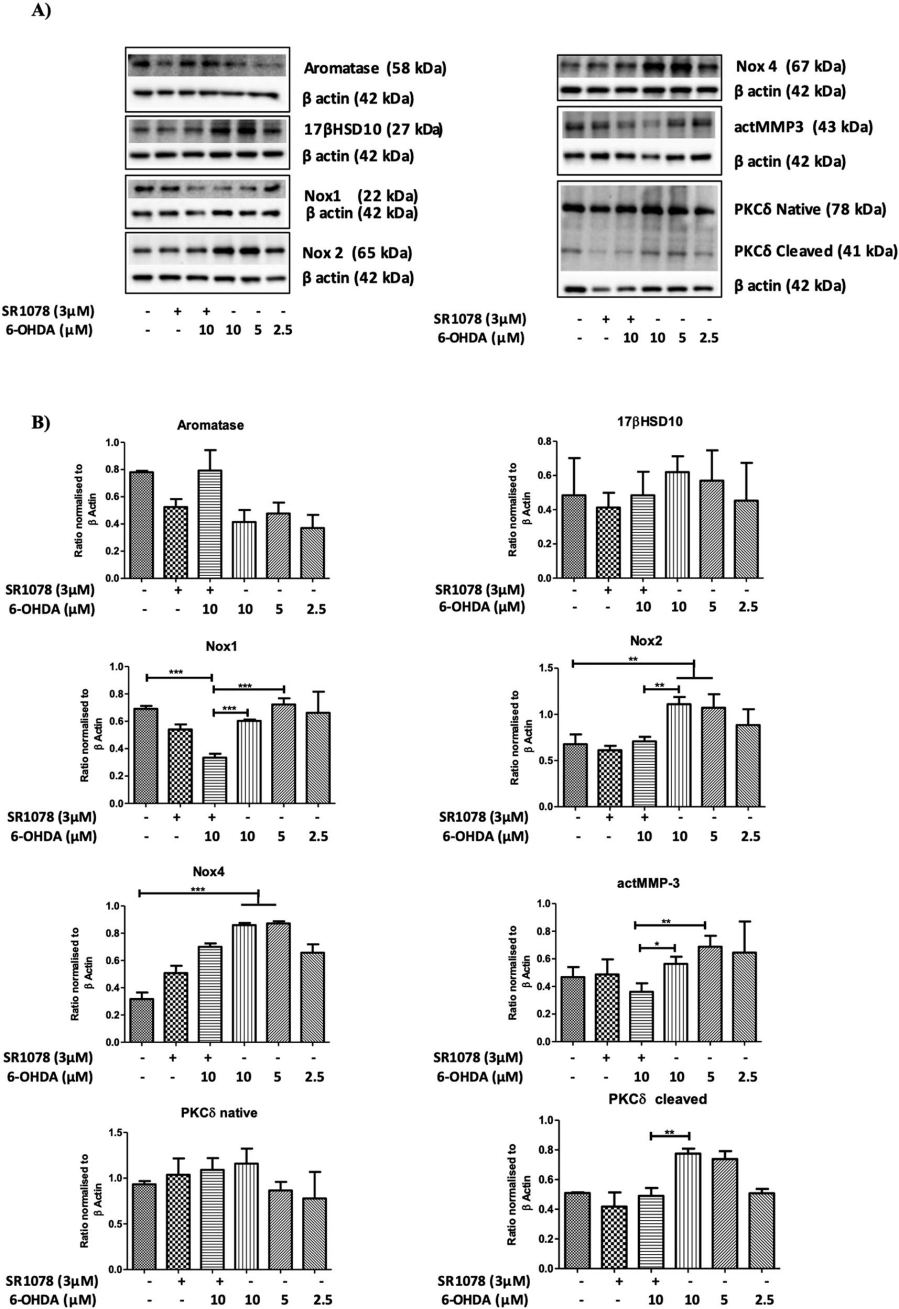
Effects of 6-OHDA without or with pre-treatment with SR1078 on protein expression in N27 cells. Cells were exposed to 6-OHDA (2.5, 5, 10 µM) for 24 h or pre-treated with SR1087 (3 µM) for 24 h prior to exposure to 6-OHDA (10 µM). **A** Representative western blot for Aromatase (58 kDa), 17βHSD10 (27 kDa), Nox1 (22 kDa), Nox2 (65 kDa), Nox4 (67 kDa), actMMP3, the native PKCδ (78 kDa), cleaved PKCδ (41 kDa) and β actin (42 kDa). **B** Densitometric data were normalised to β actin for Aromatase, 17βHSD10, Nox1, Nox2, Nox4, actMMP3, native PKCδ and cleaved PKCδ. Data presented as mean ± SEM of 3 independent experiments, each conducted in duplicate wells. *P* values were generated by one-way ANOVA, Tukey’s post hoc tests. *P* values were considered significant when <0.05 and denoted as follows: **p < 0.01. Error bars represent standard error of the mean.

Figure 6 presents western blot quantification of key proteins associated with promoting neuroprotection or neurodegeneration in N27 cells challenged with 6-OHDA with and without pre-treatment with SR1078. For each protein SR1078 alone was without effect (Fig. 6).

While 6-OHDA alone (2.5–10 μM) appeared to reduce aromatase expression in the N27 cells and pre-treatment with SR1078 to reverse this effect, the trend was not confirmed by statistical analysis (Fig. 6A, B). Similarly, no significant effects of treatments of the cells with SR1078 or 6-OHDA with or without pre-treatment with SR1078 were seen on protein levels of 17β hydroxysteroid dehydrogenase 10 (17β HSD10) (Fig. 6A, B).

Protein quantification of nicotinamide adenine dinucleotide phosphate (NADPH) oxidase catalytic isoforms, Nox1, Nox2 and Nox4, are shown in Fig. 6A, B. For Nox1, neither 6-OHDA (2.5, 5 and 10 μM) nor SR1078 (3 μM) alone had any significant effect on protein levels. However, pre-treatment with SR1078 (3 μM) significantly reduced Nox1 levels induced by subsequent exposure to 5 and 10 μM 6-OHDA (*P* ≤ 0.001) (Fig. 6A, B). For Nox 2, 6-OHDA (5 and 10 μM) exposure did significantly increase protein levels, which were significantly attenuated by pre-treatment with SR1078 (3 μM) (*P* ≤ 0.01) (Fig. 6A, B). Exposure of N27 cells to 6-OHDA (5 and 10 μM) also significantly increased protein levels for Nox4 (Fig. 6A, B). Although pre-treatment with SR1078 showed a trend to reverse this effect of 6-OHDA on Nox4, it did not reach statistical significance (Fig. 6A, B).

For active-matrix metalloprotease-3 (actMMP-3), neither 6-OHDA nor SR1078 alone altered protein levels in the N27 cells. However, when cells were treated with SR1078 (3 μM) prior to 6-OHDA exposure, a significant decrease in actMMP-3 protein levels compared to 6-OHDA alone and untreated controls was observed (*P* < 0.5) (Fig. 6A, B), suggesting that SR1078 could block cleavage of MMP-3.

Protein levels of both native and cleaved protein kinase Cδ (PKCδ) are presented in Fig. 6A, B. While SR1078 or 6-OHDA with or without pre-treatment with SR1078 had no clear statistically significant effect on the native protein, levels of cleaved PKCδ were significantly increased after exposure of cells to 6-OHDA (5, 10 μM) (Fig. 6A, B). This effect was completely blocked by pre-incubation of N27 cells with SR1078 (3 μM) (*P* ≤ 0.01).

#### Measurement of mitochondrial ROS production

**Fig. 7:**
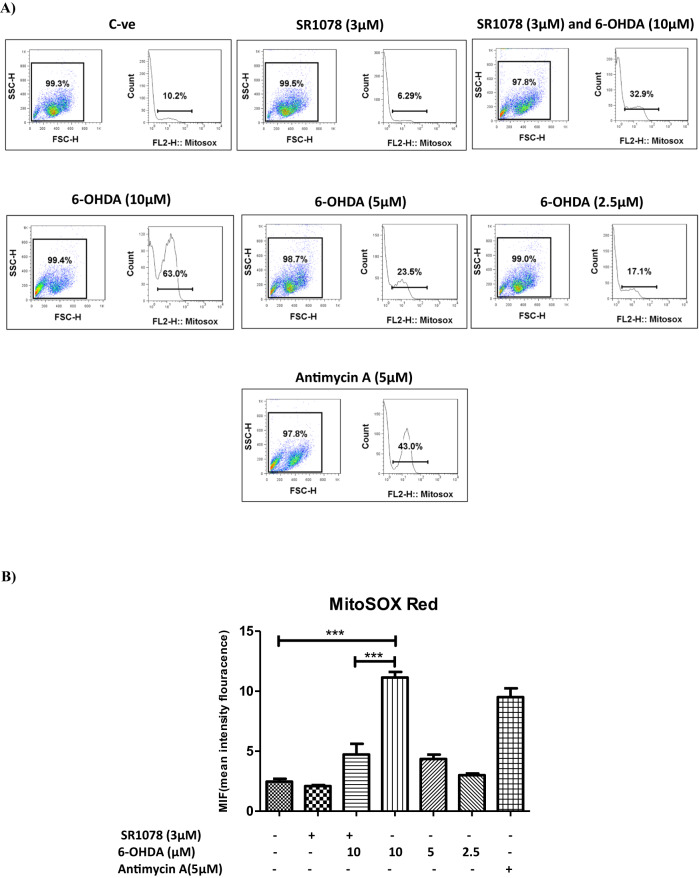
The effect of pre-treatment with SR1078 prior to 6-OHDA exposure on mitochondrial ROS production by N27 cells. Measurement of mitochondrial ROS production using MitoSOX Red flow cytometery in the N27 cells treated with increasing concentration of 6-OHDA (2.5, 5, 10 µM) for 24 h and in cells pre-treated with or without SR1078 (3 µM) for 24 h prior to exposure to 6-OHDA (10 µM). Positive control is Antimycin A (complex III inhibitor). **A** Data presented by histograms in term of the mean fluorescent intensity of MitoSox red and percentage of MitoSOX red positive cells. **B** Data presented as mean ± SEM of 3 independent experiments. *P* values were generated by one-way ANOVA, Turkey’s post hoc tests. ***P < 0.001. Error bars represent standard error of the mean.

Exposure of N27 cells to 6-OHDA (2.5, 5, 10 μM) was associated with a dose-dependent increase in mitochondrial ROS production reaching almost 5 times the level seen in the untreated controls at 10 μM 6-OHDA. This effect was markedly attenuated by pre-treatment of the cells with SR1078 (3 μM) (*P* ≤ 0.001), which alone had no effect on ROS levels (Fig. 7). Antimycin A (complex III inhibitor) served as a positive control for mitochondrial ROS production.

#### Annexin-V and Propidium Iodide flow cytometry as a measure of apoptosis

**Fig. 8:**
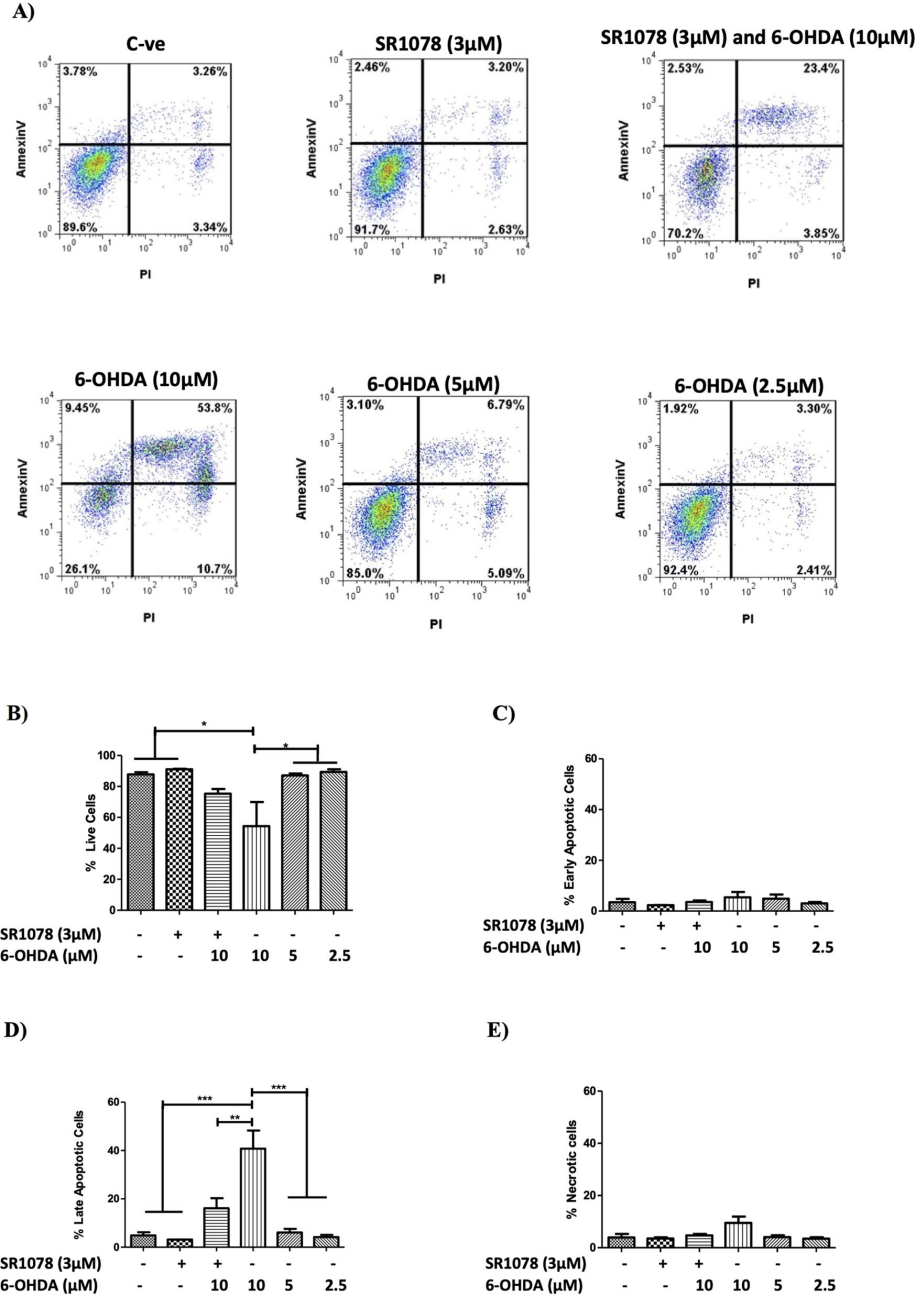
Flow cytometry analysis of annexin-V and propidium iodide (PI) staining in N27 cells pre-treated with or without SR1078 prior to 6-OHDA exposure. **A** Annexin -V and propidium iodide staining in N27 cells exposed to 6-OHDA (2.5, 5, 10 µM) for 24 h or pre-treated with SR1087 (3 µM) for 24 h prior to exposure to 6-OHDA (10 µM). Data presented as mean ± SEM of 3 independent experiments for (**B**) Live cells (double negative), (**C**) Early apoptotic (low expression of Annexin V), (**D**) Apoptotic (Annexin V positive) and (**E**) Necrotic (double positive). *P* values were generated by one-way ANOVA, Tukey’s post hoc tests. P values were considered significant when <0.05 and denoted as follows: *p < 0.05, **p < 0.01, ***p < 0.001. Error bars represent standard error of the mean.

Staining the cells with annexin-V and propidium iodide was next used to investigate the proportion of cells that were live, or in early apoptosis, late apoptosis or necrosis. Exposure to 6-OHDA (10 μM) resulted in a significantly greater proportion of cells in the late apoptotic stage (Fig. 8A, D) and a reduction in the proportion of live cells (Fig. 8A, B) compared with the untreated control cells. Treatment of the cells with SR1078 alone had no effect on the cellular profile, whereas pre-treatment with SR1078 (3 μM) prior to 6-OHDA (10 μM) exposure caused a significant increase in the population of live cells (Fig. 8A, B) (*P* < 0.05) and significant reduction in the late apoptotic cell population (*P* ≤ 0.01) (Fig. 8A, D) compared to cells exposed to 6-OHDA alone.

The results of our investigations into the potential mechanisms of action RORα/γ ligands in our cellular model of PD are summarised in Supplementary Table 3.

## Discussion

This study is the first to identify sex differences in RORA mRNA and protein levels in the SNpc of the human post-mortem brain and how its expression is differentially altered in male and female subjects with a neuropathological diagnosis of PD. As RORA expression in the CgCx was unaltered by sex or PD, we conclude that our observations have selectivity for the SNpc. We also provide direct evidence that RORA has neuroprotective properties in an in vitro model of PD employing dopaminergic neurones, representing the population in the parkinsonian SNpc that degenerates.

In humans and other animal species, neurochemical and structural sex differences are found throughout the brain, including the SNpc and nigrostriatal dopaminergic (NSDA) pathway, which are thought to have clinical as well as functional consequences^19,37,38^. These include contributions to sex differences in the prevalence, age at onset, symptomatology, progression, pathology and therapeutic responsiveness in PD, as well as numerous other brain disorders^38,39^, but the underlying mechanisms are largely unknown. Given our in vitro evidence of RORA’s neuroprotective properties, coupled with the sexually differentiated expression of SNpc RORA, we hypothesise that the greater expression of RORA in the normal SNpc in women offers resilience to neurodegenerative processes relative to men, whereas the increase in RORA expression in the male, but not female, parkinsonian SNpc represents a neuroprotective adaptation to the disease, whilst this may already be at its maximum capacity in females. RORA is thus a protective and sex discriminating factor in PD. Others have reported neuroprotective properties of RORA using in vivo and in vitro non-PD animal models. For example, disruption in the RORA gene in the mouse leads to profound loss of cerebellar neurones, cerebellar atrophy and ataxia^40^. Neurones cultured from RORA deficient mice also indicate neuroprotective functions of RORA, whereas primary mouse cortical cultures over-expressing the human RORA are protected against a variety of stressors, including hypoxia^29^ and amyloid beta^32^. Our human and in vitro findings extend this to PD, highlighting a potential role for RORA in the observed sex differences in the normal and degenerating NSDA pathway and also as a novel therapeutic target for PD.

The factors that influence sex differences in RORA expression in the human SNpc remain to be determined, but the prevailing sex steroid hormone environment is likely to play a role. Specifically, the RORA gene has sex steroid hormone receptor binding domains in its promoter region leading to up-regulation by oestradiol, via oestrogen receptor alpha, and down regulation by the non-aromatisable, dihydrotestosterone via the androgen receptor^30^. In support of the latter, frontal cortical levels of RORA were lower in males with autism^35^, a condition consistently associated with a male bias and a hyperandrogenic state^39^. Of note, clinical and experimental studies show that sex hormones also have significant influences in PD. Hence, circulating levels of oestradiol in females were protective factors, whereas circulating androgens were not, and may even exacerbate the condition, illustrating that sex hormones contribute to sex bias in PD^22^. However, the underlying mechanisms are unclear. In view of the fact that RORA gene and protein expression is significantly greater in the SNpc of the control group of women compared with men, we propose that RORA represents a mechanism through which sex hormones play a role in relative resilience (females) or susceptibility (males) to PD. It has been reported that testosterone levels in male PD subjects is reduced^41,42^, so it should be considered that this could lift the androgen-mediated suppressive influence on RORA expression, thereby accounting for its elevation in our male, not female, PD group. However, given the evidence from the present study, and work by others of the neuroprotective effects of RORA^39^, we favour the view that in male PD the increase in RORA is a neuroprotective response. In accord with this, it is known that adaptive mechanisms occur within the surviving neurones of the degenerating SNpc-striatal pathway, which compensate for neuronal loss and maintain function until up to 80% of dopaminergic neurones are lost and motor symptoms are evident^1^.

In order to investigate directly potential neuroprotective effects of RORA, we established an in vitro model of PD employing the dopaminergic N27 neuronal cell line challenged with 6-OHDA, which is a well-established DA-selective neurotoxin widely used in experimental PD both in vivo and in vitro^43^. Here, we took advantage of the recently characterised RORα/γ agonist (SR1078) and antagonist (SR1001), which, to our knowledge, have not yet been investigated in experimental models of neurodegeneration. These experiments demonstrated that 24-hour exposure of the N27 cells to increasing concentrations of 6-OHDA caused a dose-dependent increase in RORA expression, whilst their treatment with the RORA antagonist, SR1001, prior to challenge with 6-OHDA exacerbated neuronal loss and reduced survival. These findings provide evidence that dopaminergic neurones inherently express RORA, and an increase in the endogenous synthesis of RORA provides neuroprotection on neurodegenerative challenge. Thus, they provide direct evidence to support our proposal that the increase in RORA expression in the SNpc of our male PD group relative to controls is, indeed, likely to represent a protective mechanism in the surviving dopaminergic neurones.

Complementary to our results with the RORA antagonist, we have shown that exogenous application of the RORα/γ agonist, SR1078, in our in vitro PD model consistently protects the N27 neurones against 6-OHDA induced degeneration, and subsequent investigations focused on the underlying mechanisms. Initially, we considered RORA gene transcriptional targets, 17βHSD10, which is known to play an important role in maintaining mitochondria health and integrity^44^, and the enzyme aromatase, which catalyses the central conversion of androgens to oestradiol^25^. The latter property is of interest, because local synthesis of oestradiol in the brain plays a pivotal role in neuroprotection by increasing oestradiol levels at sites of brain injury^10^, including our in vivo 6-OHDA rat model of PD^22^. We hypothesised that protein levels of these enzymes would be raised in the N27 cells which had SR1087 treatment prior to 6-OHDA challenge relative to those without SR1078 pre-treatment. However, we found no significant effect to suggest that SR1078 can protect via enhancing expression of aromatase or 17βHSD10 in N27 cells. We have, however, identified important points in the intracellular signalling cascades involved in oxidative stress-induced apoptosis and impairment of mitochondrial function whereby SR1078 may act to limit cell death in our N27 model of PD, and which are known to be central to PD pathology^37^, as well as to mechanisms of 6-OHDA-induced cell death in experimental PD^43^.

Over activation of NADPH oxidases (NOXes) are major sources of cellular reactive oxygen species (ROS) and oxidative stress in neurodegenerative diseases^45,46^, and appear to be involved in the pathogenesis of PD^47^ . Here, we demonstrate that Nox 2 and Nox 4 levels are significantly elevated in N27 cell cultures after exposure to 6-OHDA treatment and SR1078 pre-treatment significantly blocked this rise in Nox2 and Nox4. This indicates that inhibition of NOX enzymes could be a promising target in PD and supports other studies using MPP+ as the DA selective toxin in N27 cells^47^. Given the evidence that ROS production is an early and essential step in the apoptotic signalling cascade^48^ and ROS levels in post-mortem PD brains were reported to be elevated^49,50^, we then investigated ROS production in the N27 cells and found that this was significantly increased by 6-OHDA exposure, which was blocked by pre-treatment with SR1078. This corroborates evidence from a study involving over expression of human RORA in mouse primary cortical neurones^26^ of a role for SR1078 in inhibition of ROS production. This effect could be partly due to a reduction in actMMP-3 as current thoughts suggest that activation of actMPP-3 is involved in the neurodegenerative process in PD and was found to increase ROS production^51^. In support of the role of actMMP-3 in PD pathology, it was found be expressed in Lewy bodies in the SN of post-mortem PD brains^52^ and its immunoreactivity was increased in TH positive dopaminergic neurons in the SN of mice injected with MPTP. On the other hand, actMMP-3 knockout mice showed reduced dopaminergic cell death in MPP+ model of PD^53^. Moreover, actMMP-3 has been reported to induce cleavage of α-synuclein with a consequent increase in aggregation of α-synuclein and neurotoxicity^54,55^. Experimentally, rats injected with 6-OHDA also showed increased expression of actMMP-3^54^ leading to increase mitochondrial ROS production^56^. The current study showed a significant reduction in actMMP3 in N27 cells pre-treated with SR1078 prior to 6-OHDA compared to untreated cells exposed to 6-OHDA alone, further supporting the neuroprotective prosperities of SR1078.

The pro-oxidant sensitive kinase, PKCδ, is an important substrate for caspase 3 and a prominent player in oxidative stress induced apoptosis^57^. It is highly expressed in dopaminergic nigral neurones and its proteolytic cleavage by caspase 3 has been reported to be a key event in 6-OHDA induced dopaminergic neuronal death in cellular and animal models of PD, including rescue of N27 cells and TH+ mesencephalic neurones by PKCδ siRNA and loss of function induced by PKCδ gene mutations^58^. Our results demonstrate that pre-treatment of the N27 cells with the RORα/γ agonist prior to 6-OHDA challenge significantly blocked the cleavage of PKCδ compared to cells exposed to 6-OHDA without SR1078 pre-treatment. Furthermore, pre-treatment with SR1078 also countered the 6-OHDA induced increase caspase 3/7, an indicator of apoptosis. This evidence for neuroprotective effects of SR1078 via apoptotic mechanisms was extended using Annexin V flow cytometry. This showed a significant increase in late apoptotic cells and a reduction in live cells in the N27 cells exposed to 6-OHDA compared with unexposed cells, whereas SR1078 pre-treatment increased the population of live cells and decreased the size of the late apoptotic population. In support of the role of apoptosis in neuronal death in PD, apoptotic neurons were identified in the SNpc of PD subjects^59–61^. Collectively, our results identify SR1078 as a potential therapeutic target for PD. Supplementary Fig. 1 summarises the findings of the current study.

In conclusion, this is the first demonstration that there are inherent sex differences in RORA mRNA and protein levels in the human SNpc, and that these are differentially altered in male and female PD subjects. This leads us to conclude that RORA may have significance in both a physiological and neuropathological context, with consequences for the sex differences that characterise PD. Having validated a replicable in vitro system to serve as a translational model of PD, we have shown that the RORα/γ agonist, SR1078, has significant neuroprotective properties acting via various mechanisms. These include inhibition of actMMP-3 and reduction of mitochondrial ROS production, a reduction in Nox1 and Nox2, a reduction of caspase 3 and cleavage of PKCδ, all of which would contribute to the observed reduction of oxidative stress and apoptosis in our dopaminergic cell line challenged with 6-OHDA. This is the first study to link RORA to PD in the human brain and the first to investigate the recently characterised RORα/γ ligands in experimental models of neurodegeneration. Collectively, they support a protective and sexually differentiating role for RORA in PD and highlight the translational importance of RORA in PD.

## Material and methods

### Human post-mortem brain samples

Tissue was collected from human post-mortem brains of male and female subjects displaying late-stage pathology-confirmed PD (Braak α-synuclein stage 5 and 6, *n* = 14)^62^ and from age- and sex-matched control subjects (*n* = 11). The use of brain tissue was approved by the Parkinson’s UK Brain Bank (PUKBB) Ethical Review Panel for all experiments and all subjects provided written informed consent while living to donate their brains upon death. The average age of the control subjects was 81 years (range 73–89 years), and the average age of the patients with PD was 81 years (range 78–84 years). The full details of the cases used in the study are provided in Supplementary Table 1. Brain tissue was removed from the body of the donor less than 24 h post-mortem and immediately dissected following the PUKBB standardized dissection protocol^63^. One hemisphere was cut into blocks that were snap frozen using isopentane pre-chilled on dry ice and stored at −80 °C. Samples containing the SNpc were subsequently processed for mRNA and protein extraction, as described below. Samples were also collected from the anterior cingulate cortex (CgCx) as a control region which suffers little cell loss at late stages of the disease, but is still affected by α-synuclein pathology^62^. The other hemisphere was immersed in paraformaldehyde for 4 weeks until fixed. Tissue blocks were then dissected from the fixed brain and embedded in paraffin wax.

### Quantification of RORA gene expression

For each SNpc and CgCx tissue sample mRNA was extracted and cDNA prepared as previously described^64^. HPLC purified primers and probe (Integrated DNA Technologies) were used for the detection of RORA by real time quantitative PCR (**probe** 5′/56-FAM/cagtttttc/ZEN/aatttttaccttttcttgcagcca/3IABkFQ/-3′), (**primer 1**,5′-ttttctgcatttgtactgatgtca-3′), (**primer 2**,5′-ctcggtgattcttctgtaggac-3′). Specificities of the chosen sequences were checked using nucleotide basic local alignment search tool (BLAST) searches on the NCBI web site^65^. Reactions to quantify the expression of RORA utilized Brilliant® II qPCR master mix with low carboxyrhodamine ROX (Agilent TechnologiesK Ltd., Edinburgh, UK) and were run (95 °C for 10 min, then 95 °C for 30 s, 55 °C for 30 s and 72 °C for 30 s for 60 cycles) using a Stratagene Mx3005P real-time PCR system (Agilent Technologies) running MxPro software (v4.10, Stratagene). Separate reactions for the two reference genes (GAPDH, β-actin) were run alongside the experimental samples on each plate. Reactions were carried out using the following cycling protocol: 95 °C for 10 min, then 60 cycles with 3-step program (95 °C for 30 s, 55 °C for 30 s, and 72 °C for 30 s). Fluorescence data collection was made during the annealing (55 °C) step. In each plate, a negative control for each PrimeTime™ qPCR assay containing no cDNA template was included. To analyse the data for relative gene expression, the comparative CT (^ΔΔ^CT) method was used^66^, normalizing gene expression to the geometric mean expression of the two reference genes (GAPDH and β actin)^67^.

### Quantification of RORA protein expression in PD and controls

Western blot analysis with chemiluminescent detection was performed to quantify the RORA and aromatase protein levels in SNpc and CgCx^40^. Brain tissue from each area of interest (30 mg) was homogenized using a Tissue Tearor homogenizer (Model 985370-395, BioSpec Products Inc., USA) in 300 μL RIPA lysis buffer (Sigma) with Pierce^TM^ protease inhibitor mixture^68^. The samples were kept on ice during the extraction procedure and for another 30 min after homogenization, after which the lysate was centrifuged at 10,000 g for 10 min at 4 °C. The supernatant was removed and stored at −80 °C until protein quantification using the Bradford assay (Sigma) on the day that the western blot analysis was performed.

Chemiluminescent western blot analysis was performed^68^ on 20 µg protein dissolved in Laemmeli sample buffer (Bio-Rad). Before loading, samples were incubated at 95 °C for 10 min. Following electrophoresis, proteins were transferred to polyvinylidene fluoride (PVDF) membranes (pore size, 0.2 μm) using a Trans-Blot Turbo semi-dry transfer system (Bio-Rad, Hemel membranes were then equilibrated in PBST (phosphate buffered saline with 0.2% Tween-20) before blocking with 4% BSA for 1 h. After blocking, the membranes were incubated with a primary antibody (see Supplementary Table 2) overnight at 4 °C on a shaker. The membranes were washed 4 times each for 15 min before incubating then with a horseradish peroxidase (HRP)-conjugated secondary antibody (see Supplementary Table 2) for 1 h at room temperature. The membranes were then washed with TBST 4 times each for 15 min at room temperature and were developed with, MA, USA) for 1 min. Images were taken by a chemiluminescence imaging system (GeneGnome XRQ, SYNGENE, Cambridge, UK). Protein bands were quantified using densitometry analysis software (ImageJ-win64). All blots were processed in parallel and derived from the same experiment.

### Cell culture experiments

#### In vitro experimental PD

To complement the findings from the post-mortem human study and to investigate the potential neuroprotective properties of RORα, we developed an in vitro cell culture model of PD utilising the N27 human cell line (generous gift from Dr. Nabil Hajji, Division of Brain Sciences, Imperial College London) to represent dopaminergic neurones, as they express the dopamine (DA) synthesising enzyme, tyrosine hydroxylase (TH) and exhibit typical neuronal morphology. The N27 cells are challenged with 6-hydroxydopamine (6-OHDA), which is commonly used both in vitro and in vivo in experimental PD due to its relative selective toxicity for dopaminergic neurones^37^. Neurodegeneration and potential neuroprotective capacity and underlying mechanisms of RORα/γ ligands were then assessed using a range of viability and survival assays and measures of intracellular factors known to be involved in these processes, as detailed below. All experiments were conducted in duplicate wells for 3 independent experiments.

#### Cells culture and treatment paradigm

##### N27 neuronal cell line

The rat dopaminergic 1RB_3_A_N27_ (N27) cell line is an immortalized clone of rat mesencephalic dopaminergic neurons produced by transfecting foetal mesencephalic cells with the plasmid vector pSV3 _neo_, which carries the LTa gene from the SV_40_ virus^69^. The N27 cells have been characterized and found to have a morphology similar to dopamine neurones and to express many markers, including TH, characteristic of dopaminergic neurons, which make them a good candidate cell line for PD modelling in vitro^70^. Our investigations showed that these cells lack the Y chromosome, so it is considered female sex.

N27 cells were maintained in 75 cm^2^ cell culture flask (Corning, NY, USA) in RPMI medium (Gibco, Life Technologies, Paisley, UK) supplemented with 5% foetal bovine serum (unless otherwise stated) (Sigma, UK), 2% L-Glutamine and 1% penicillin-streptomycin (all from Sigma-Aldrich Poole, Dorset) at 37 °C in 5% CO_2_ humidified atmosphere. N27 was passaged every 3 to 4 days, when confluence reached approximately 75%. N27 cells were used to maximum passage number 12 and all experiments were performed at passage number 5 to 12.

##### Passaging and seeding of the N27 cells

Cells were passaged once they reached 75% confluency (around 3–4 days). First, the flask was washed twice with 5 ml sterile medium at 37 °C (Dulbecco’s Phosphate Buffered Saline (DPBS) (Sigma, UK) for N27 cells or Hanks’ Balanced Salt solution (HBSS) (Sigma, UK) in order to remove any trypsin inhibitors that present in the foetal calf serum in the medium. Cells then were detached by using 5 ml 0.25% trypsin (Sigma, UK), incubated for 2 min after which the flask was tapped firmly against the palm of the hand to detach all adherent cells. Then the suspension was collected followed by washing the flask with medium and adding that to the collected suspension to stop the trypsin activity. The cell suspension then was centrifuged at 1200 × *g* for 5 at 23 °C using Labofuge 400 R, Heraeus, UK. The cell pellet was re-suspended in 1 ml of warm complete medium RPMI for N27 supplemented with heat inactivated filter-sterilized FBS (10% and 5%, respectively) or 1% Double-stripped serum, 2% L-glutamine (Sigma, UK), 1% penicillin streptomycin (Sigma, UK)), and then diluted to 15 ml. The number of viable cells was estimated by mixing 10 µl of cell suspension with 10 µl of trypan blue stain (Sigma, UK). The viable, unstained cells within 10 μl volume of this mixture were then counted using haemocytometer. Cell density was then adjusted by appropriate dilution with complete medium to achieve a final density of 314 cells/mm^2^ or 154 cells/mm^2^ when added to the wells, as described for each experiment. Plates then were maintained in the incubator for 24 h to allow the cells to attain normal morphology before starting the experiment. The remaining un-used cells were used to repopulate a new 75 cm^2^ flask as described above.

##### Preparation of 6-OHDA and stimulation of N27

A stock solution of 6-OHDA (MW: 250.09) at 10 mM was prepared by dissolving the contents of a 5 mg vial 6-OHDA (Sigma-Aldrich Poole, Dorset) in 2 ml deionised water containing 0.01% ascorbic acid as an antioxidant stabilizer; 120 µl aliquots were stored at −20 °C for later use. All Eppendorf tubes were covered with foil to protect 6-OHDA from light. After 24 h’ incubation of plated cells, medium was replaced with fresh medium containing the calculated doses of 6-OHDA for 24 h’ incubation.

##### SR1078 and SR1001

RORα/γ agonist (SR1078; mw 431.25) and antagonist (SR1001; mw 477.4) were both purchased from TocrisBioscience (Avonmouth, Bristol, United Kingdom). Stock solutions of 10 mM were prepared by diluting 10 mg of SR1078 and SR1001 in 2.3188 ml and 2.0947 ml, respectively, of sterile filtered DMSO (TocrisBioscience, Avonmouth, Bristol, United Kingdom). The stock of each was stored at -20 °C as light-protected 50 µL aliquots. Desired concentrations were calculated by dividing the initial stock concentration by the desired concentrations to get the dilution factor.

##### Positive controls for viability assays and MitoSox red assay

The positive control for the viability assays, Staurosporine (Sigma, UK), is a multiple kinases inhibitor that was originally isolated from the bacterium *Streptomyces staurosporeus* in 1977, and it stops the cell cycle at G1^71^. Staurosporine was used as a positive control for viability assays at a concentration of 1 μM. Initial concentration 1 mM in DMSO was diluted in the medium with dilution factor 1:100 and 100 µl was added to 96 well plate and 500 µl to 24 well plate. The positive control for the MitoSox red flow cytometry is Antimycin A^72^, which is a potent inhibitor of the mitochondrial electron transport chain^73^. Cells were treated with 5 µM by adding 5 µl of 1 mM stock solution to 995 µl of medium.

##### Testing the neuroprotective properties of SR1078

For testing the neuroprotective potential of the RORAα/γ agonist and antagonist, cells were plated in multiwell plates (96 well plate at density of 157 cells/mm^2^ for MTS and caspase 3/7 activity, 24 well plate at density of 157 cells/mm^2^ for LDH assay, in 6 well plate at density of 157 cells/mm^2^ for western blot) for 24 h in a phenol red free medium supplemented with 1% double charcoal stripped foetal calf serum (a precaution to eliminate potential steroidogenic/neuroprotective activity of these factors that would interfere with 6-OHDA toxicity as well as effect of potential neuroprotective agents), 2% L-Glutamine and 1% penicillin-streptomycin (all from Sigma-Aldrich Poole, Dorset). After the initial 24 h’ incubation, cells were exposed to a second 24 h incubation period with medium containing SR1078 (3, µM) or SR1001 (5, 10, 15, 20 µM). This was followed by a third 24 h incubation period in which the medium was replaced with fresh medium containing a concentration of 6-OHDA (10 µM) that had previously been shown to cause a sub-maximal destruction of the cells. Controls comprised (i) a set of cells that received normal culture medium with no drugs added (negative control) and (ii) a set of cells that received normal medium with no additives during the first and second incubation and medium containing Staurosprotine during the third incubation (positive control).

##### Viability and survival assays

After the experimental endpoint, the viability of the N27 cells was established in two ways using the 3-(4,5-dimethylthiazol-2-yl)-5-(3-carboxymethoxyphenyl)-2-(4-sulfophenyl)-2H-tetrazolium, inner salt (MTS) and Lactate Dehydrogenase (LDH) Assays^29^ along with measures of apoptosis and Reactive Oxygen Species (ROS) production, as described below.

#### MTS assay

For the MTS assay, the kit CellTiter 96® Aqueous One Solution Cell Proliferation Assay from Promega (Madison, USA) was used according to the manufacturer’s instructions^29,74,75^. Saturosporine (Sigma) was used as a positive control. Plates were read at 490 nm using a spectrophotometer (VersaMax Microplate Reader, Molecular Devices, CA, USA).

#### LDH assay

For the LDH assay the kit CytoTox 96® Non-Radioactive Cytotoxicity Assay from Promega, (Madison, USA) was used according to the manufacturer’s instruction. Both released and total LDH were measured, and the results were expressed as a ratio of the released to the total LDH. Plates were read at 490 nm using a spectrophotometer (VersaMax Microplate Reader, Molecular Devices, CA, USA).

#### Caspase 3/7 activity for measuring apoptosis

To measure apoptosis in the cell cultures Cell Metre™ Caspase 3/7 Activity Apoptosis Assay Kit (AAAT Bioquest, California, USA) was used, where TF2-DEVD-FMK is a fluorescent indicator for caspase 3/7 activity, according to the manufacturer’s instructions. Fluorescence intensity was measure using a fluorescence plate reader (Glomax multidetection system, Promega, Madison, USA) at Ex/Em = 600 nm and data were presented as relative fluorescent units.

#### MitoSOX™ Red staining for Fluorescence-Activated Cell Sorting (FACS)

MitoSOX™ Red reagent is a live-cell dye that at the low concentration of 1 µM, is highly selective to mitochondrial ROS^76^. For detection of ROS production in N27 cells, MitoSOX™ Red mitochondrial superoxide indicator (Invitrogen, USA) was used according to the manufacturer’s instructions. FACS then was performed using BD Biosciences FACSCalibur, Midland, ON, Canada, selecting FL2 channel with the peak around Ex/Em 510/580 nm. Data were analysed using the software, FlowJo, LLC.

#### Annexin V for Fluorescence-Activated Cell Sorting (FACS)

In experiments investigating the influence of the RORα/γ agonist on 6-OHDA toxicity on N27 cells we used an eBioscience™ Annexin V-FITC Apoptosis Detection Kit (ebioscience, USA)^77^ to determine the type of cell death. FACS then was performed (BD Biosciences FACSCalibur, Midland, ON, Canada) using FL2 filter. Data were analysed using the software, FlowJo, LLC.

#### Western blot analysis

At the experimental endpoint, medium was removed and N27 cells were washed two times with ice-cold PBS. Cells were collected by scraping the cells with non-pyrogenic sterile cell scraper (Corning Costar) in 300 μL ice-cold PBS. The collected cells were centrifuged for 5 min at 4 °C, 5 × 1000 g using Biofuge fresco, Heraeus, UK. PBS was removed and the cell pellet was suspended in 70 μL RIPA buffer (50 mM Tris- HCl, pH 8.0, with 150 mM sodium chloride, 1.0% Igepal CA-630 (NP-40), 0.5% sodium deoxycholate, and 0.1% sodium dodecyl sulfate, Sigma) supplemented with PierceTM Protease Inhibitor Mini Tablets (Thermoscientific) which contain protease- inhibitor cocktail (AEBSF, Aprotinin, Bestatin, E64, EDTA, Leupeptin, Pepstatin A), for each 10 ml of RIPA buffer, one 30 mg tablet of protease inhibitor was dissolved. After 30 min incubation of the cells in RIPA buffer supplemented with the protease inhibitor in ice, the lysate was centrifuged for 10 min at 4 °C, 5 × 1000 g using Biofuge fresco, Heraeus, UK. The supernatant was removed and stored at −20 for later protein quantification by western blot analysis, as described above for human samples. Supplementary Table 2 summarizes the primary and secondary antibodies, blocking and incubation conditions applied for western blotting in the cell culture experiments.

### Statistical analysis

Data for PD and controls were analysed using the Mann–Whitney *T* test. The power of the study was calculated based on calculation of the effect size^78^ applying Cohen’s d values^79,80^, which indicated that the *P* value of our data was substantial. For cell culture experiments, all experiments were performed as three independent replicates. Data were analysed and graphed using GraphPad Prism version 5. Since normality testing showed the data was non-parametric paired comparisons were made using the Mann–Whitney test and multiple comparisons used the Kruskal–Wallis one-way ANOVA by ranks (*H*). When ANOVA indicated a significant difference, *post hoc* Tukey’s multiple comparison test was used to compare all treatment groups to the control group. All tests were two-tailed and the significance level for all tests was taken to be *P* < 0.05. Unless otherwise stated, statistical significance is indicated in the figure legends using the following system: *p* < 0.05 (*), *p* < 0.01 (**), *p* < 0.001 (***), *p* < 0.0001 (****).

### Reporting summary

Further information on research design is available in the Nature Research Reporting Summary linked to this article.

## Supplementary information

supplementary materials

Reporting summary

Supplementary figures legends

## Data Availability

The data supporting the findings of this study are available upon request from the corresponding author.
