# Supplementary material for: Neuroprotective role for RORA in Parkinson’s disease revealed by analysis of post-mortem brain and a dopaminergic cell line
Source: NPJ Parkinsons Dis. 2023 Jul 27;9:119. doi: 10.1038/s41531-023-00563-4 (PMC10374904; doi:10.1038/s41531-023-00563-4)
Supplement: Supplementary file 1 — supplementary materials [file 41531_2023_563_MOESM1_ESM.pdf]

Supplementary materials

Tables

Supplementary Table 1. Demographic data for PD and control cases

Control (C)	Age	Sex	αSYN score	PMI	PD Case	Age	Sex	Onset	Duration	αSYN score	PMI
C1	71	F	0	17	PD1	87	F	76	12	6	?
C2	78	F	0	23	PD2	85	F	67	18	6	14
C3	84	F	0	11	PD3	82	M	65	18	6	14
C4	77	M	0	17	PD4	77	M	67	10	6	6
C5	81	M	0	19	PD5	80	M	60	19	6	16
C6	90	M	0	12	PD6	83	M	73	9	6	10
C7	94	M	0	10	PD7	82	M	72	11	6	10
C8	81	M	0	8	PD8	75	M	50	25	6	15
C9	80	M	0	28	PD9	75	M	67	8	6	3
C10	89	F	0	22	PD10	79	M	59	20	5	22
C11	66	F	0	12	PD12	79	M	55	24	5	19
					PD13	78	F	59	19	6	?
					PD14	84	F	84	11	5	16
Mean	81.0			16.3	Mean	80.1		63.1	17.1		12.3
SD	8.2			6.3	SD	3.8		9.3	7.3		5.8

PMI = post-mortem interval; αSYN score = α-synuclein Braak stage; C = control; PD = Parkinson's disease.

Supplementary Table 2. Antibodies Used for Western Blotting.

Antigen	Molecular weight	Blocking conditions	Primary antibody	Primary antibody incubation conditions	Secondary antibody	Secondary antibody incubation conditions
**RORA**	67 kDa	4% BSA in PBS-T for 1 hour at RT	Rabbit polyclonal anti-RORA (Santa Cruz Biotechnology Inc.)	1:1000 in 4% BSA in PBS-T overnight at 4°C	HRP-conjugate anti-rabbit goat antibody (Sigma, UK)	1:2000 in4% BSA in PBS-T for 1 hour at RT
**Aromatase (CYP19)**	50 kDa	4% BSA in PBS-T for 1 hour at RT	Mouse monoclonal anti-CYP19 (Santa Cruz Biotechnology Inc.)	1:500 in4% BSA in PBS-T overnight at 4°C	HRP-conjugate anti-mouse goat antibody (cell signalling)	1:2000 in4% BSA in PBS-T for 1 hour at RT
**β Actin**	42 kDa	4% BSA in PBS-T for 1 hour at RT	Mouse monoclonal anti-β Actin (Sigma, UK)	1:3000 in 4% BSA in PBS-T overnight at 4°C	HRP-conjugate anti-mouse goat antibody (cell signalling)	1:2000 in4% BSA in PBS-T for 1 hour at RT
**17βHSD10 (ERAB)**	27 kDa	4% BSA in PBS-T for 1 hour at RT	Rabbit monoclonal anti ERAB antibody (Abcam, Cambridge, UK)	1:10,000 in 4% BSA in PBS-T over night at 4°C	HRP-conjugate anti-rabbit goat antibody (Sigma, UK)	1:2000 in 4% BSA in PBS-T for 1 hour at RT
**Nox1**	65 kDa	4% BSA in PBS-T for 1 hour at RT	Rabbit monoclonal to Nox1 (Abcam, Cambridge, UK)	1:5000 in 4% BSA in PBS-T over night at 4°C	HRP-conjugate anti-rabbit goat antibody (Sigma, UK)	1:2000 in 4% BSA in PBS-T for 1 hour at RT

**Nox2**	60 kDa	4% BSA in PBS-T for 1 hour at RT	Rabbit monoclonal to Nox2 (Abcam, Cambridge, UK)	1:5000 in 4% BSA in PBS-T over night at 4°C	HRP-conjugate anti-rabbit goat antibody (Sigma, UK)	1:2000 in 4% BSA in PBS-T for 1 hour at RT
**Nox4**	67 kDa	4% BSA in PBS-T for 1 hour at RT	Rabbit monoclonal to Nox4 (Abcam, Cambridge, UK)	1:2000 in 4% BSA in PBS-T over night at 4°C	HRP-conjugate anti-rabbit goat antibody (Sigma, UK)	1:2000 in 4% BSA in PBS-T for 1 hour at RT
**Protein Kinase Cδ (PKCδ)**	Native 78 kDa<br>Cleaved 42 kDa	4% BSA in PBS-T for 1 hour at RT	Rabbit monoclonal to PKCδ (Abcam, Cambridge, UK)	1:5000 in 4% BSA in PBS-T over night at 4°C	HRP-conjugate anti-rabbit goat antibody (Sigma, UK)	1:2000 in 4% BSA in PBS-T for 1 hour at RT
**Matrix Metalloprotease (MMP)3-cleaved**	40 kDA	4% BSA in PBS-T for 1 hour at RT	Rabbit polyclonal toMMP3 - cleaved (Sigma, UK)	1:1000 in 4% BSA in PBS-T over night at 4°C	HRP-conjugate anti-rabbit goat antibody (Sigma, UK)	1:2000 in 4% BSA in PBS-T for 1 hour at RT

Incubations and blocking conditions used for western blotting.

Abbreviations: BSA, bovine serum albumin; **PBS-T**, Phosphate buffered saline containing 0.2% Tween-20; **HRP**, horseradish peroxidase; **RT**, room temperature.

Protein level of neuroprotective/neurodegenerative factors			Effect of 6-OHDA alone vs control	Influence of pre-treatment with SR1078 on effect of 6-OHDA*
Associated with promoting neuroprotection	aromatase		↔	↔
	17βHSD10		↔	↔
Associated with promoting neurodegeneration	Nox 1		↔	↓ vs 6-OHDA alone; ↓ vs negative control
	Nox 2		↑ (Significant at 10 µM only)	Totally blocked
	Nox 4		↑ (dose dependent)	↔
	actMMP3		↔	Protein level suppressed below untreated control, suggesting SR1078 could block cleavage of actMMP3 at higher 6-OHDA concentration
	Mitochondrial ROS		↑↑ (dose dependent)	Totally blocked
	PKCδ native		↔	↔
	PKCδ cleaved		↑ (dose dependent)	Totally blocked
	Apoptosis (Annexin V & PI)	Live	↓to 50%	Blocked
		Early apoptotic	↔	↔
		Late apoptotic	↑ (significant at 10 µM only)	Blocked
		Necrotic	↔	↔

Table 3 Summary of the characterized mechanism of action of SR1078

Summary of the influence of the RORα/γ agonist, SR1078, on protein expression (determined by western blot analysis) of factors implicated as upstream or downstream mediators of RORα-dependent neuroprotection and factors associated with promoting neurodegeneration in an *in vitro* PD model of 6-OHDA toxicity in the dopaminergic N27 cell line. * SR1078 alone had no effect. ↔ (no effect), ↑ (increased), ↓ (decreased).

Supplementary Figure 1

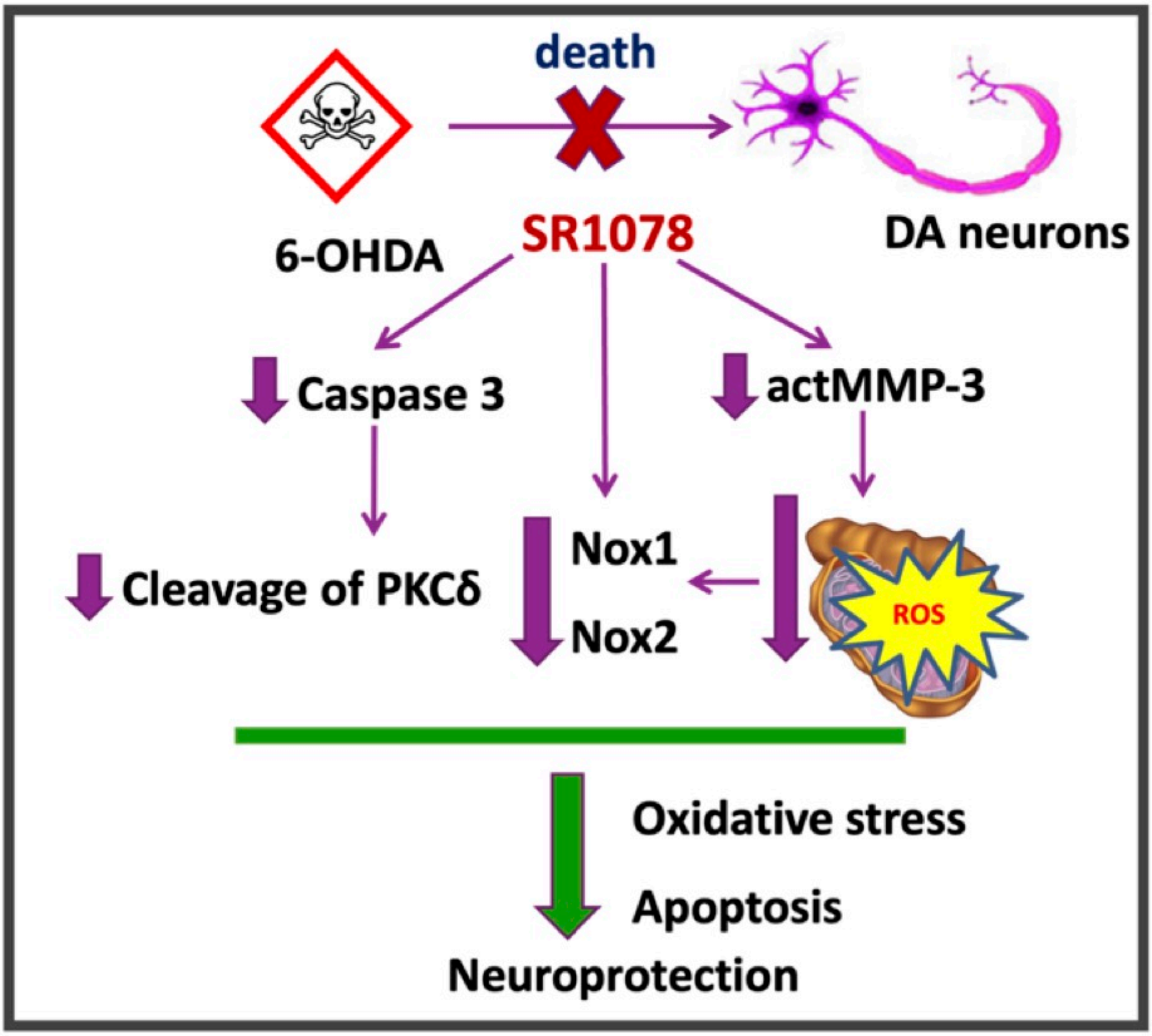

Western Blotting - uncropped blots

Figure 1

RORA male CgCx

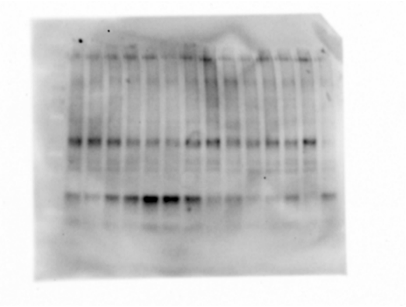

Beta actin male CgCx

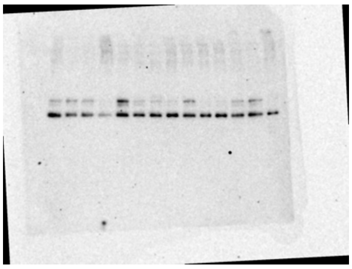

RORA female CgCx

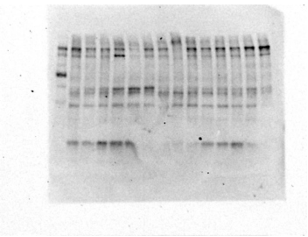

Beta actin female CgCx

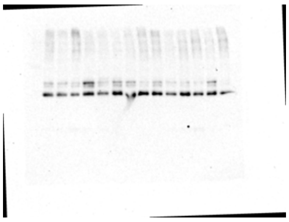

Figure 5

RORA expression in N27 with increasing doses of 6-OHDA

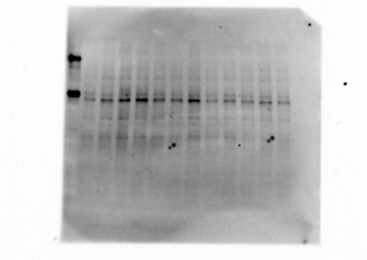

Its beta actin
