# Supplementary material for: Neuroprotective role for RORA in Parkinson’s disease revealed by analysis of post-mortem brain and a dopaminergic cell line
Source: NPJ Parkinsons Dis. 2023 Jul 27;9:119. doi: 10.1038/s41531-023-00563-4 (PMC10374904; doi:10.1038/s41531-023-00563-4)
Supplement: Supplementary file 2 — Reporting summary [file 41531_2023_563_MOESM2_ESM.pdf]

nature portfolio

Corresponding author(s): Felwah Saad Al-Zaid

Last updated by author(s): 2/7/2023

Reporting Summary

Nature Portfolio wishes to improve the reproducibility of the work that we publish. This form provides structure for consistency and transparency in reporting. For further information on Nature Portfolio policies, see our Editorial Policies and the Editorial Policy Checklist.

Statistics

For all statistical analyses, confirm that the following items are present in the figure legend, table legend, main text, or Methods section.

n/a	Confirmed	
☐	☒	The exact sample size (n) for each experimental group/condition, given as a discrete number and unit of measurement
☐	☒	A statement on whether measurements were taken from distinct samples or whether the same sample was measured repeatedly
☐	☒	The statistical test(s) used AND whether they are one- or two-sided<br>*Only common tests should be described solely by name; describe more complex techniques in the Methods section.*
☐	☒	A description of all covariates tested
☐	☒	A description of any assumptions or corrections, such as tests of normality and adjustment for multiple comparisons
☐	☒	A full description of the statistical parameters including central tendency (e.g. means) or other basic estimates (e.g. regression coefficient) AND variation (e.g. standard deviation) or associated estimates of uncertainty (e.g. confidence intervals)
☒	☐	For null hypothesis testing, the test statistic (e.g. F, t, r) with confidence intervals, effect sizes, degrees of freedom and P value noted<br>*Give P values as exact values whenever suitable.*
☒	☐	For Bayesian analysis, information on the choice of priors and Markov chain Monte Carlo settings
☒	☐	For hierarchical and complex designs, identification of the appropriate level for tests and full reporting of outcomes
☐	☒	Estimates of effect sizes (e.g. Cohen's d, Pearson's r), indicating how they were calculated

Our web collection on statistics for biologists contains articles on many of the points above.

Software and code

Policy information about availability of computer code

Data collection	*Provide a description of all commercial, open source and custom code used to collect the data in this study, specifying the version used OR state that no software was used.*
Data analysis	*Provide a description of all commercial, open source and custom code used to analyse the data in this study, specifying the version used OR state that no software was used.*

For manuscripts utilizing custom algorithms or software that are central to the research but not yet described in published literature, software must be made available to editors and reviewers. We strongly encourage code deposition in a community repository (e.g. GitHub). See the Nature Portfolio guidelines for submitting code & software for further information.

Data

Policy information about availability of data

All manuscripts must include a data availability statement. This statement should provide the following information, where applicable:

- Accession codes, unique identifiers, or web links for publicly available datasets
- A description of any restrictions on data availability
- For clinical datasets or third party data, please ensure that the statement adheres to our policy

Data are available upon request.

Research involving human participants, their data, or biological material

Policy information about studies with human participants or human data. See also policy information about sex, gender (identity/presentation), and sexual orientation and race, ethnicity and racism.

Reporting on sex and gender	yes, gender was mentioned in the manuscript as samples were separated based on sex either male or female.
Reporting on race, ethnicity, or other socially relevant groupings	non
Population characteristics	Male and females controls and Parkinson's disease patients, mean age 81 years, full characteristics are described in supplementary table 1.
Recruitment	From Parkinson's UK Brain Bank
Ethics oversight	Parkinsons UK Brain Bank

Note that full information on the approval of the study protocol must also be provided in the manuscript.

Field-specific reporting

Please select the one below that is the best fit for your research. If you are not sure, read the appropriate sections before making your selection.

☒ Life sciences ☐ Behavioural & social sciences ☐ Ecological, evolutionary & environmental sciences

For a reference copy of the document with all sections, see nature.com/documents/nr-reporting-summary-flat.pdf

Life sciences study design

All studies must disclose on these points even when the disclosure is negative.

Sample size	was based on availability of samples then we measured the effect size to check validity of our data.
Data exclusions	non
Replication	data done in replicates.
Randomization	Random
Blinding	not relevent

Reporting for specific materials, systems and methods

We require information from authors about some types of materials, experimental systems and methods used in many studies. Here, indicate whether each material, system or method listed is relevant to your study. If you are not sure if a list item applies to your research, read the appropriate section before selecting a response.

Materials & experimental systems

n/a	Involved in the study	
☐	☒	Antibodies
☐	☒	Eukaryotic cell lines
☒	☐	Palaeontology and archaeology
☒	☐	Animals and other organisms
☒	☐	Clinical data
☒	☐	Dual use research of concern
☒	☐	Plants

Methods

n/a	Involved in the study	
☒	☐	ChIP-seq
☐	☒	Flow cytometry
☒	☐	MRI-based neuroimaging

Antibodies

Antibodies used	All antibodies are well described as requested in supplementary table 2 and 3.
Validation	See supplementary table 2 and 3.

Eukaryotic cell lines

Policy information about cell lines and Sex and Gender in Research

Cell line source(s)	N27 cell lines was a generous gift from Dr. Nabil Haji in Brain sciences Division in college of medicine, Imperil college London.
Authentication	N27 cells was authenticated by appearance as it was having normal morphology for dopaminergic like cell line and by western blot analysis that confirmed expression of the TH (tyrosine hydroxylase) which is marker for deopamainergic cells. PCR analysis showed that it doesn't express Y gene so it is female in origin.
Mycoplasma contamination	All cell lines tested negative for mycoplasma.
Commonly misidentified lines (See ICLAC register)	*Name any commonly misidentified cell lines used in the study and provide a rationale for their use.*

Flow Cytometry

Plots

Confirm that:

☒ The axis labels state the marker and fluorochrome used (e.g. CD4-FITC).

☒ The axis scales are clearly visible. Include numbers along axes only for bottom left plot of group (a 'group' is an analysis of identical markers).

☒ All plots are contour plots with outliers or pseudocolor plots.

☒ A numerical value for number of cells or percentage (with statistics) is provided.

Methodology

Sample preparation	N27 cells were seeded at density of 157 cells/ mm2 in 24 well plates. At experimental endpoint, medium was collected in round-bottom polystyrene tubes (Corning costar, thermoscientific, UK). Cells were detached with 0.25% trypsin, after being washed twice with PBS. The collected cells were centrifuged at 4oC for 5 minutes at 1200 xg using Labofuge 400R, Heraeus, UK. Medium was gently aspirated and the cell pellet was re-suspended in 1 ml PBS for a second centrifugation. Then the PBS was gently aspirated and the cells were re-suspended in 300µl of MitoSox Red (1µM), protected from light and placed in the incubator, at 37oC 5% CO2 for 15 minutes.
Instrument	FACS then was performed (BD Biosciences FACSCalibur, Midland, ON, Canada) using FL2 filter
Software	Data were analysed using the software, FlowJo, LLC.
Cell population abundance	Cells were counted as in sample preparation.
Gating strategy	*Describe the gating strategy used for all relevant experiments, specifying the preliminary FSC/SSC gates of the starting cell population, indicating where boundaries between "positive" and "negative" staining cell populations are defined.*

☒ Tick this box to confirm that a figure exemplifying the gating strategy is provided in the Supplementary Information.
