# Supplementary material for: Neuroprotective role for RORA in Parkinson’s disease revealed by analysis of post-mortem brain and a dopaminergic cell line
Source: NPJ Parkinsons Dis. 2023 Jul 27;9:119. doi: 10.1038/s41531-023-00563-4 (PMC10374904; doi:10.1038/s41531-023-00563-4)
Supplement: Supplementary file 3 — Supplementary figures legends [file 41531_2023_563_MOESM3_ESM.docx]

**Supplementary Figures Legends**

**Supplementary Figure 1. Schematic summary for the mechanism of action of SR1078 in protecting N27 cells against 6-OHDA**

Pre-treating N27 cells with SR1078 prior to 6-OHDA was found to protect the cells through many mechanisms. It acts through inhibition of actMMP-3 which subsequently leads to reduction in mitochondrial ROS production. Reduced mitochondrial ROS leads to decrease in Nox1, Nox2 and Nox3 which subsequently lead to reduced generation of oxidative stress. SR1078 reduces caspase 3 activity and proteolytic cleavage of PKCδ.
